# Botulinum toxin for the treatment of dystonia and pain in corticobasal syndrome

**DOI:** 10.1002/brb3.1182

**Published:** 2019-05-09

**Authors:** Elisa Unti, Sonia Mazzucchi, Rosanna Calabrese, Giovanni Palermo, Eleonora Del Prete, Ubaldo Bonuccelli, Roberto Ceravolo

**Affiliations:** ^1^ Neurology Unit Ospedale Apuano Massa Italy; ^2^ Neurology Unit, Department of Medical Specialties Azienda Ospedaliero Universitaria Pisana Pisa Italy; ^3^ Neurology Unit, Department of Clinical and Experimental Medicine University of Pisa Pisa Italy

**Keywords:** botulinum toxin, corticobasal syndrome, dystonia, pain

## Abstract

**Background:**

Dystonia is a key symptom in corticobasal syndrome (CBS), and upper limb dystonia is the most common phenotype. Dystonia‐associated pain is frequently reported and can be disabling, with poor benefit from oral treatments.

**Aims of the Study:**

To investigate the role of botulinum toxin A (BoTNA) in the treatment of dystonia and associated pain in CBS.

**Methods:**

Ten consecutive patients with a clinical diagnosis of probable CBS and dystonia with/without associated pain were treated with BoTNA every 3 months. Treatment efficacy was assessed during the first follow‐up visit, three months after the first injection, by means of caregiver impression (CI), evaluation of muscle tone with the Ashworth scale (AS), severity of pain measured with the visual analog scale (VAS).

**Results:**

Nine subjects underwent at least three treatments, four patients discontinued for progressive reduction in efficacy or disease progression, five patients are ongoing with good response, and one completed the 10th treatment. No local or systemic side effects were reported, and levodopa equivalent daily dose remained unchanged in most cases during the observational period. Significant improvement of AS was recorded (from 2.9 ± 0.7 to 2.0 ± 0.5, *p* = 0.003). CI ranged from mild to moderate benefit. All patients reported efficacy on pain, with a significant reduction of VAS score (from 7.7 ± 1.7 to 1.7 ± 0.7 in the Pain group, *p* = 0.016).

**Conclusions:**

Our study confirms safety, efficacy, and tolerability of BoTNA in the treatment of dystonia associated with CBS. Local treatment should be considered as a valid alternative to oral treatment modulation mainly in the presence of associated pain.

## Abstract 2

Dystonia is a key symptom in corticobasal syndrome (CBS), and upper limb dystonia is the most common phenotype. The role of botulinum toxin A (BoTNA) in the treatment of dystonia and associated pain in CBS has been not investigated so far Our study confirms safety, efficacy, and tolerability of BoTNA in the treatment of dystonia associated to CBS and related pain in 10 subjects.

## 1 INTRODUCTION

Corticobasal degeneration (CBD) is a rare neurodegenerative disease included in the group of tauopathies. According to pathologically proven cases and current diagnostic criteria (Alexander et al., 2014; Armstrong et al., 2013; Stamelou, Alonso‐Canovas, & Bhatia, 2012), the disease may present with different phenotypes, mainly as corticobasal syndrome (CBS), but also as progressive supranuclear palsy syndrome, frontotemporal dementia, and nonfluent primary progressive aphasia. The term CBS is currently preferred to CBD (Stamelou et al., 2012) when referring to cases without anatomopathological confirm. Probable CBS is defined by the asymmetric presentation of two motor symptoms (limb rigidity/akinesia, limb dystonia, limb myoclonus) associated with two nonmotor features (orobuccal/limb apraxia, cortical sensory deficit, alien limb phenomena). Mean disease duration is 6.6 years (range 2–12.5 years). Limb rigidity is present in the majority of cases during the overall course of the disease (85%), whereas dystonia affects a variable percentage of subjects (Armstrong et al., 2013). The onset of dystonic symptoms usually occurs during the first 2 years from diagnosis but can be delayed up to 4 years. The dystonic pattern is variable, and upper limb is the most commonly affected, whereas in minority of cases, cervical dystonia, blepharospasm, and lower limb dystonia can be observed (Stamelou et al., 2012). Upper limb dystonia can present as a posture typically defined “dystonic clenched fist,” characterized by flexion and adduction of arm, forearm, wrist, and metacarpophalangeal joints, with the extension of interphalangeal joints (Cordivari, Misra, Catania, & Lees, 2001). Associated pain is frequently reported and can be disabling.

Therapeutical strategies in CBS are limited and mainly rely on the treatment of motor symptoms. Parkinsonism can benefit from levodopa in a minority of cases whereas dystonia is usually not responsive, with dose increase often unsuccessful and poorly tolerated. Benefit on dystonic symptoms from nondopaminergic treatments such as benzodiazepines, anticholinergics, or baclofen is rare and transient, often burdened by side effects (Kompoliti et al., 1998; Marsili, Suppa, Berardelli, & Colosimo, 2016).

Efficacy of local treatment with botulinum toxin A (BoTNA) in the treatment of painful dystonia occurring in Parkinson’s disease (PD) and parkinsonisms has been reported (Bruno, Fox, Mancini, & Miyasaki, 2016; Bruno et al., 2018; Pacchetti et al., 1995); however, no controlled studies are available, in particular regarding dystonia and associated pain in atypical parkinsonisms (AP).

In pathologically proven cases of CBD, clinical efficacy of BoTNA on dystonia was reported, but only a minority of patients were treated, without taking into account specific outcome measures (Kompoliti et al., 1998). Improvement of dystonic posturing, hand gripping, and hand hygiene was reported in two CBD patients, and relief of associated pain was hypothesized but described only in patients affected by different subtypes of AP (Müller, Wenning, Wissel, Seppi, & Poewe, 2002). Cordivari and colleagues investigated the effect of BoTNA in the treatment of “dystonic clenched fist” associated with CBD, PD, and complex regional pain syndrome (CRPS). All three CBD patients included had dystonia‐associated pain and reported significant benefit even if no functional improvement was noticed. Pain relief was reported also in PD and CRPS (Cordivari et al., 2001).

## 2 AIMS OF THE STUDY

The aim of our study was to investigate the role of BoTNA in the treatment of dystonia and pain in CBS patients, searching for possible predictors of good and prolonged response to treatment to improve the selection of patients.

## 3 METHODS

Consecutive patients referring to the Movement Disorders Centre of Pisa with a diagnosis of probable CBS, according to current diagnostic criteria (Armstrong et al., 2013), and presenting with dystonia were included in the study. Informed consent was obtained, and all procedures performed were in accordance with the ethical standards of the institutional research committee and with the 1964 Helsinki declaration and its later amendments or comparable ethical standards.

After signing informed consent, clinical data were collected. The Ashworth scale (AS, score 0–4) was used to evaluate muscle tone. Even if the scale is validated for spasticity, it has been used in previous studies to assess muscle tone in dystonia because more specific tools are not available. Presence and severity of pain associated with dystonic posture were measured with the visual analog scale (VAS, 0–10) and recorded during all the observational period. After clinical and electrophysiological evaluation, patients were treated with BoTNA (T0). Selection of muscles depended on clinical phenotype and neurophysiological recordings (Table 1). All patients were injected with abobotulinumtoxinA (Dysport, dilution: 500 UI in 2.5 ml of saline). Treatment efficacy was evaluated during the follow‐up visit performed 3 months after the first injection (T1) taking into account both patient’s and caregiver’s impression (CI). CI was expressed by means of a specific scale ranging from 0 to 3 (0 = no efficacy, 1 = mild benefit, 2 = moderate benefit, and 3 = significant benefit). The CI and VAS scores were obtained at T1 but they were referred to the period of maximum efficacy of the treatment. The Ashworth score was measured at T1. An intermediate visit was not performed between T0 and T1 to reduce patients’ discomfort.

**Table 1:** Clinical details before (T0) and after the first treatment (T1)

Patient	Age	Disease duration (months) at T0	Type of dystonia	Injected muscles	BoTNA total dose (UI)	VAS score T0	VAS score T1	Ashworth Score T0	Ashworth score T1	Functional effect	Caregiver impression
1	73	72	Left arm	Left biceps, triceps, brachioradialis, flexor digitorum profundus	500	8	0	3	2	No	2
2	76	36	Neck	Trapezium, splenium capitis, sternocleidomastoid	320	0	0	3	2	No	2
3	79	14	Left arm	Left biceps, brachioradialis	300	8	0	3	2	No	2
4	82	36	Right arm	Right biceps, triceps, brachioradialis, flexor digitorum profundus	500	0	0	4	3	No	0
5	69	96	Right arm	Right biceps, triceps, brachioradialis	350	0	0	3	2	No	0
6	69	48	Left arm	Left Biceps, brachioradialis, flexor digitorum profundus	380	9	1	3	2	No	1
7	68	36	Left arm	Left flexor digitorum superficialis, lumbricalis	80	8	0	1	1	Yes	3
8	80	24	Left arm	Left biceps, triceps, brachioradialis, flexor digitorum profundus	240	6	1	3	2	No	2
9	76	24	Right arm	Right biceps, brachioradialis	100	5	0	3	2	No	1
10	76	20	Right arm	Right biceps, brachioradialis	200	10	1	3	2	No	1

Note

BoTNA: botulinum toxin A; VAS: visual analog scale.

Data were analyzed with SPSS version 24, using nonparametric statistical tests. The significance level in all statistical tests was set to 0.05. Wilcoxon signed‐rank test was used to compare median levodopa equivalent daily dose (LEDD), VAS, and AS before and after treatment. Correlations were assessed using Spearman’s rank correlation coefficient.

## 4 RESULTS

Ten consecutive patients (females, mean age 74.8 ± 4.9 years) were included in the study (Table 1). Dystonia affected the upper limb in nine patients, and one had cervical dystonia. In all cases, levodopa treatment at T0 was ongoing with unsatisfactory results (mean LEDD 593.5 ± 276.6, median 675.0). At T0, mean injected dose of abobotulinumtoxinA was 297.0 ± 145.8 UI, disease duration was 40.6 ± 25.5 months, and mean AS was 2.9 ± 0.7. Seven patients complained of pain associated with dystonia, with mean T0 VAS score of 5.4 ± 4.0 considering the whole population and 7.7 ± 1.7 in the group reporting pain (Pain group). Correlations between AS and VAS score were not observed at T0 (*p* = 0.253).

Patients were followed up and injected every 3 months up to 30 months. No local or systemic side effects were reported. One patient discontinued after the second treatment due to lack of efficacy, and 9 subjects underwent at least three treatments. Four patients discontinued, respectively, after 3rd (*n* = 1), 4th (*n* = 1), and 6th (*n* = 2) injection for progressive reduction in benefit or disease progression. Five patients are ongoing with good response, and one subject completed the 10th treatment.

LEDD remained unchanged in most cases during the observational period (mean LEDD at last treatment 631.0 ± 178.0, median 625.0), without significant differences between first and last treatment (*Z* = −0.736, *p* = 0.461). Levodopa dose increase was limited by side effects in three cases (hypotension, behavioral problems) and progression of dysphagia in one case. All subjects reported poor benefit on motor symptoms from oral treatment.

At T1, mean AS was 2.0 ± 0.5, with a significant reduction of median values (median T0: 3.0 median T1: 2.0; *Z* = −3.0, *p* = 0.003; Table 2). CI ranged from mild to moderate benefit, with mean score 1.4 ± 0.9 in the whole population and 1.7 ± 0.7 in the Pain group. Improvement was observed on passive movements, hand hygiene, and quality of sleep. Functional improvement of the hand was reported only in the subject with AS = 1 at T0.

**Table 2:** Mean clinical scores before (T0) and after the first treatment (T1)

	T0	T1	*p*
Visual analog scale (0–10)			
Study population	5.4 ± 4.0	0.3 ± 0.5	0.016
Pain group	7.7 ± 1.7	0.4 ± 0.5	
Ashworth score (0–4)	2.9 ± 0.74	2.0 ± 0.5	0.003
Caregiver impression (0–3)			
Study population		1.4 ± 0.9	
Pain group		1.7 ± 0.7	

All patients complaining of pain at T0 reported a sustained benefit, with mean VAS score at T1 of 0.3 ± 0.5 in the whole population and 0.4 ± 0.5 in the Pain group. The VAS reduction was statistically significant (median T0: 7.0 median T1: 0, *Z* = −2.414, *p* = 0.016). In three cases, remission of pain was not complete at T1, but further benefit was observed after the following treatments. Effect on pain remained stable over time even in those patients who discontinued treatment due to progressive loss of motor benefit.

No correlation was found between duration of treatment, expressed as number of treatments, and VAS score (*p* = 0.847), AS (*p* = 0.161), or disease duration at T0 (*p* = 0.849). CI at T1 showed correlation with the number of treatments (*p* = 0.005).

## 5 DISCUSSION

The main aim of our study was to investigate efficacy of BoTNA in the treatment of dystonia and associated pain in CBS patients.

Our data confirm safety of BoTNA in CBS and its better tolerability with respect to oral treatment.

As previously reported (Kompoliti et al., 1998), dopaminergic therapy is unsatisfactory both on parkinsonism and on dystonia in the majority of patients and it is frequently associated with side effects (30% cases in our population). Nevertheless, local treatment with BoTNA is rarely offered to CBS patients.

In our cohort, it was not possible to demonstrate functional improvement, possibly limited by mean disease duration at T0 (40.6 months); however, similar results were reported in previous studies as part of the natural history of the disease. According to our results, local treatment can be helpful in a multimodal way, first of all by reducing hypertonia (*p* = 0.003), facilitating hygiene and passive movements. Muscle tone reduction observed could have been underestimated due to low specificity of the Ashworth scale and lack of an intermediate evaluation between T0 and T1.

Cognitive impairment of treated patients could have affected perception of efficacy. CI scale was used to add an objective measure of treatment efficacy and to evaluate real impact on quality of life. Mild to moderate benefit reported by caregivers, more pronounced in the Pain group, supports reliability of patients’ impression.

Significant pain reduction was reported by all seven subjects (*p* = 0.016), with complete remission after the first injection in four patients. Pain is a disabling symptom affecting quality of life, and oral drugs are often burdened by side effects and cognitive worsening. Efficacy of BoTNA on dystonic pain in CBS has been reported, but validated outcome measures are not currently available. In a recent retrospective study, pain response was evaluated using the Clinical Global Impression scale (Bruno et al., 2016), whereas VAS was used to report pain severity in dystonia related to advanced PD (Bruno et al., 2018).

Cognitive impairment in our patients could have limited the understanding of the VAS, and the subjective relief on pain could be part of a placebo response; however, in the same subjects, previous oral treatments were ineffective and there was always agreement between patient and caregiver impression on pain improvement.

We observed that even if duration of global treatment efficacy was variable among subjects, in all cases benefit on pain remained stable during the observational period and after discontinuation of BoTNA injections. In our cohort, pain associated with dystonia was not reported by all subjects and did not show a direct correlation with muscle tone, fixed posture, or disease duration. We hypothesized that efficacy of BoTNA on this symptom could be the result of a combined peripheral and central action, restoring possible dysfunctions of pain networks.

Secondary aim of our study was to identify predictors of good and prolonged response to improve selection of injected patients. According to our data, it is not possible to recognize statistically significant predictors, even if a positive CI suggests a prolonged efficacy expressed as total number of treatments (*p* = 0.005).

Our study has several limits, mainly the limited sample size and lack of a control group. Moreover, selection of patients was made on the basis of a clinical diagnosis of probable CBS.

To our knowledge, this is the largest CBS population treated with BoTNA and evaluated with outcome measures focused on muscle tone and pain.

## 6 CONCLUSION

The present study confirms the safety, efficacy, and tolerability of BoTNA in upper limb and cervical dystonia associated with CBS.

Our results underline the importance to consider local treatment as a valid alternative to oral treatment modulation, mainly in patients complaining of pain associated with dystonia.

## CONFLICT OF INTEREST

All the authors report no conflict of interest.
